# Exploring the origins of neurodevelopmental proteasomopathies associated with cardiac malformations: are neural crest cells central to certain pathological mechanisms?

**DOI:** 10.3389/fcell.2024.1370905

**Published:** 2024-07-12

**Authors:** Virginie Vignard, Alban-Elouen Baruteau, Bérénice Toutain, Sandra Mercier, Bertrand Isidor, Richard Redon, Jean-Jacques Schott, Sébastien Küry, Stéphane Bézieau, Anne H. Monsoro-Burq, Frédéric Ebstein

**Affiliations:** ^1^ Nantes Université, CHU Nantes, CNRS, INSERM, l’institut du thorax, Nantes, France; ^2^ CHU Nantes, Department of Pediatric Cardiology and Pediatric Cardiac Surgery, FHU PRECICARE, Nantes Université, Nantes, France; ^3^ Nantes Université, CHU Nantes, INSERM, CIC FEA 1413, Nantes, France; ^4^ Nantes Université, CNRS, INSERM, l’institut du thorax, Nantes, France; ^5^ CHU Nantes, Service de Génétique Médicale, Nantes Université, Nantes, France; ^6^ Faculté des Sciences d'Orsay, CNRS, UMR 3347, INSERM, Université Paris-Saclay, Orsay, France; ^7^ Institut Curie, PSL Research University, CNRS, UMR 3347, INSERM, Orsay, France; ^8^ Institut Universitaire de France, Paris, France

**Keywords:** neurodevelopmental proteasomopathies, cardiac malformations, craniofacial anomalies, neural crest cells, protein homeostasis, compensatory pathways

## Abstract

Neurodevelopmental proteasomopathies constitute a recently defined class of rare Mendelian disorders, arising from genomic alterations in proteasome-related genes. These alterations result in the dysfunction of proteasomes, which are multi-subunit protein complexes essential for maintaining cellular protein homeostasis. The clinical phenotype of these diseases manifests as a syndromic association involving impaired neural development and multisystem abnormalities, notably craniofacial anomalies and malformations of the cardiac outflow tract (OFT). These observations suggest that proteasome loss-of-function variants primarily affect specific embryonic cell types which serve as origins for both craniofacial structures and the conotruncal portion of the heart. In this hypothesis article, we propose that neural crest cells (NCCs), a highly multipotent cell population, which generates craniofacial skeleton, mesenchyme as well as the OFT of the heart, in addition to many other derivatives, would exhibit a distinctive vulnerability to protein homeostasis perturbations. Herein, we introduce the diverse cellular compensatory pathways activated in response to protein homeostasis disruption and explore their potential implications for NCC physiology. Altogether, the paper advocates for investigating proteasome biology within NCCs and their early cranial and cardiac derivatives, offering a rationale for future exploration and laying the initial groundwork for therapeutic considerations.

## 1 Introduction

Neurodevelopmental disorders (NDDs) and congenital heart diseases (CHDs) represent two major global public health challenges, affecting over 15% of children and nearly 1% of newborns worldwide, respectively (Bouma and Mulder, 2017; Liu Y. et al., 2019; Romero-Ayuso, 2021). NDDs are characterized by deficits in cognitive function and adaptive behavior (Micai et al., 2020; Hanly et al., 2021), spanning a broad spectrum of neurological conditions of varying severity including notably intellectual disability, developmental delay, autism spectrum disorders, communication and learning disorders, as well as attention deficit/hyperactivity disorder (Ismail and Shapiro, 2019). CHDs, commonly referred to as heart defects, are defined as anatomical and/or functional abnormalities of the heart or its associated great vessels present at birth. The spectrum of CHDs varies widely, encompassing mild defects with minimal clinical impact to severe and life-threatening conditions. This diversity complicates the classification of CHDs, making it both complex and challenging. NDDs represent the most prevalent morbidity among CHD patients, exerting a substantial impact on academic achievement, the transition to independence, and overall quality of life (Peyvandi et al., 2023). Herein, CHD children often demonstrate lower scores for cognition, language, attention, executive functions, as compared to their peers (Derridj et al., 2021). Despite their synergistic and cumulative nature, described as “the cumulative burden of injury,” well-known postnatal risk factors such as postnatal hypoxia/hypoperfusion, perioperative stroke, etc., account for less than 30% of adverse neurocognitive outcomes in NDD (Marelli et al., 2016; Patt et al., 2023). On the contrary, it seems that neurological defects within CHDs are primarily shaped by prenatal factors. This notion is substantiated by well-established evidence suggesting that CHDs can impact fetal brain development, leading to reduced fetal brain volume and disruptions in utero metabolic brain trajectories (Sadhwani et al., 2022; Andescavage et al., 2023; Peyvandi and Rollins, 2023). The heart and brain are indeed intricately interconnected during embryogenesis (Polat et al., 2011; Martinez-Biarge et al., 2013). For instance, certain CHDs can diminish blood oxygenation due to ineffective blood pumping by the heart, leading to insufficient oxygen and nutrients reaching the developing brain. This prenatal hypoxia can alter brain structures, potentially causing long-term neurological deficits (Wang et al., 2021). Lately, a genetic cause has also been proposed to explain the prevalence of NDDs in individuals with CHDs. This hypothesis is notably supported by a large study based on exome sequencing of CHDs patients and their parents, revealing a significantly increased burden of *de novo* variants in genes involved in both brain and heart development (Homsy et al., 2015).

## 2 Neurodevelopmental proteasomopathies associated with CHDs

Recent studies have brought to light that individuals with NDDs carrying pathogenic variants in genes related to the ubiquitin-proteasome system (UPS) may not only exhibit neurological symptoms but also present with CHDs. The UPS, a critical and conserved pathway across eukaryotes, consists of approximately 1,200 genes, enabling the covalent modification of damaged and/or unneeded proteins with ubiquitin for their subsequent degradation by 26S proteasomes (Çetin et al., 2021; Goetzke et al., 2021). Prime example of UPS dysfunction that may result in a dual phenotype of NDDs and CHDs include the recently described neurodevelopmental proteasomopathies caused by genomic alterations in proteasome genes such as *PSMD12*, *PSMC3* and *PSMC1* (Table 1).

**TABLE 1:** Clinical manifestations of well-established neurocristopathies and neurodevelopmental proteasomopathies caused by variants in the *PSMD12*, *PSMC1* and *PSMC3* genes.

OMIM	Genomic alteration(s)	Disease	Cardiac features	Neurologic features	References
188400	22q11.2 deletion (*TBX1, PITX2, COMT*)	DiGeorge syndrome (Velocardiofacial syndrome)	OFT defects	Neuronal deficits, dementia and autistic features	Wilson et al. (1992), Evers et al. (2006)
214800	*CHD7*	CHARGE syndrome	Various heart anomalies	Mental retardation	Hurst et al. (1989), Källén et al. (1999)
163950 605275	*PTPN11* *LZTR1*	Noonan syndromes	Various congenital heart defects	Mental retardation in some cases	Tartaglia et al. (2002)
105650	*RPL/RPS* (at least 16 genes)	Diamond Blackfan Anemia syndromes	Various heart malformations	Mental retardation and learning disabilities in some cases	Willig et al. (1999), Campagnoli et al. (2004), Pallanti et al. (2008)
102500	*NOTCH2*	Hajdu-Cheney syndrome	Cardiovascular anomalies, persistent ductus arteriosus, ventricular septal defect	Various neurologic symptoms	Brennan and Pauli (2001)
235730	*ZEB2*	Mowat-Wilson syndrome	Patent ductus arteriosus and/or ventricular septal defect	Intellectual disability, delayed psychomotor development	Wakamatsu et al. (2001), Ishihara et al. (2004), Evans et al. (2012)
261540	*B3GLCT*	Peters Anomaly with short-limb dwarfism	Cardiac malformations	Mental retardation	van Schooneveld et al. (1984)
261600	*PAH*	Phenylketonuria	Congenital heart disease	Hyperreflexia, kinetic tremor, slowed horizontal saccades, cognitive and behavioral abnormalities	Rouse et al. (1997), Pilotto et al. (2021)
615630	*IFT172*	Short-rib thoracic Dysplasia 10 with or without polydactyly	Various heart anomalies	Intellectual disability in some cases	Halbritter et al. (2013)
617516	*PSMD12*	Stankiewicz-Isidor syndrome	Cardiac abnormalities, including septal defects and patent ductus arteriosus	Intellectual disability and abnormal behavior, including autism	Küry et al. (2017), Khalil et al. (2018), Yan et al. (2022)
N.A.	*PSMC1*	Neurological syndrome	Muscular ventricular septal defect	Intellectual disability, dysmorphism, hearing loss	Aharoni et al. (2022)
N.A.	*PSMC3*	Neurodevelopmental disorder with type I IFN production	Ventricular or septal defects, patent ductus arteriosus, pulmonary hypertension and atresia	Developmental delay, hearing loss, intellectual disability, abnormal behavior	Ebstein et al. (2023)

**FIGURE 1:**
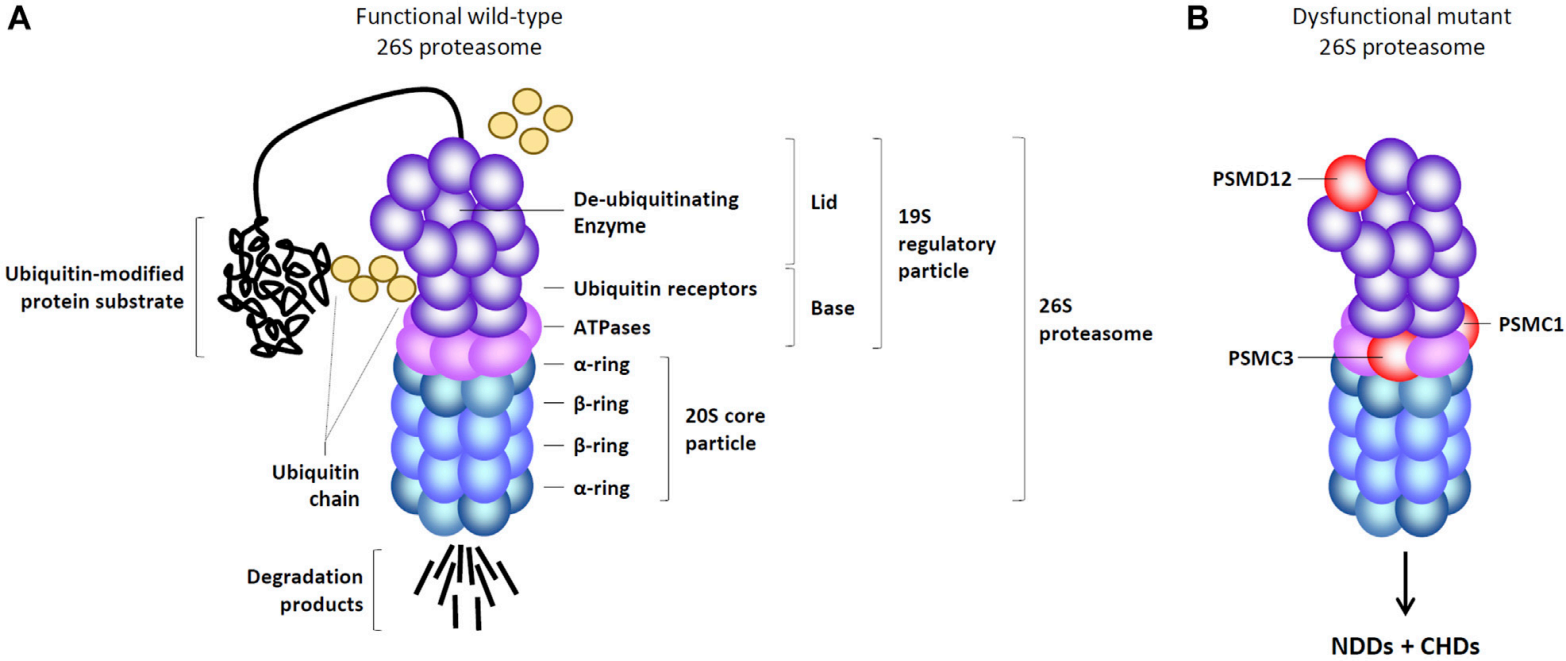
Organization of functional and mutant 26S proteasome complexes. **(A)** Functional 26S proteasomes are comprised of a 20S core particle (CP) linked to one end of the 19S regulatory particle (RP), which consists of a base and a lid. The base of the 19S RP includes six ATPase subunits (pink) and four non-ATPase subunits (purple), among which two ubiquitin receptors, as indicated. The lid of the 19S RP contains nine non-ATPase subunits (purple) including one de-ubiquitination enzyme, as indicated. The 20S CP comprises two heptameric α-rings (light blue) and two heptameric β-rings (dark blue), each of which housing three catalytic subunits. **(B)** Dysfunctional 26S proteasomes causing neurodevelopmental disorders (NDDs) associated with congenital heart diseases (CHDs) carry pathogenic variants in the PSMD12, PSMC1 and PSMC3 subunits (red), as indicated.

From a structural perspective, the 26S proteasome consists of a 20S core particle (CP) encased with a 19S regulatory particle (RP). The 19S RP comprises 19 distinct subunits, which can be subdivided into two sub-modules: the base and the lid (Bard et al., 2018; Sahu and Glickman, 2021), both susceptible to loss-of-function mutations (Figures 1A, B). The base segment comprises six diverse AAA + ATPase subunits (designated as PSMC1-6) alongside four non-ATPase subunits, namely, PSMD1, PSMD2, PSMD4, and ADRM1, respectively (Bard et al., 2018; Sahu and Glickman, 2021). The lid component consists of nine structural subunits, namely, PSMD3, PSMD6, PSMD7, PSMD8, PSMD11, PSMD12, PSMD13, PSMD14, and SEM1 (Bard et al., 2018; Sahu and Glickman, 2021). As illustrated in Figure 1A, the 20S CP is a cylindrical structure formed by stacking 28 subunits into four hetero heptameric rings: two external α-rings and two internal β-rings, encompassing the PSMA1-7 and PSMB1-7 subunits, respectively (Tanaka, 2009; Collins and Goldberg, 2017). The 19S RP detects ubiquitin-modified protein substrates via the subunits PSMD4 and ADRM1 which act as ubiquitin receptors (Deveraux et al., 1995; Husnjak et al., 2008). This recognition is followed by their subsequent de-ubiquitination by PSMD14 and their unfolding by PSMC1-6 (Verma et al., 2002; Bar-Nun and Glickman, 2012). After translocation into the 20S CP, linearized substrates are degraded into short peptides via the PSMB5, PSMB6, and PSMB7 catalytic subunits (Schmidt and Finley, 2014). Beyond maintaining protein homeostasis in the cell, the UPS plays a significant role in regulating numerous pathways by selectively targeting kinases, transcription factors, cyclins, enzymes, and/or other key cellular components for degradation (Çetin et al., 2021; Goetzke et al., 2021; Papendorf et al., 2022).

Neurodevelopmental proteasomopathies were first documented in 2017, with *PSMD12* loss-of-function mutations identified in patients presenting typical signs of syndromic intellectual disability, including speech delay and abnormal facial features (Küry et al., 2017; Khalil et al., 2018). Remarkably, in addition to neurodevelopmental features, a substantial portion of patients exhibited visceral anomalies, among which cardiac issues, particularly patent ductus arteriosus, emerged as the predominant feature (Cuinat et al., 2023). Five years later, a similar pattern of neurodevelopmental delay coupled with heart defects was observed in individuals carrying *PSMC3* loss-of-function variants (Ebstein et al., 2023). Both neurological and cardiac features were similar to those seen in patients with *PSMD12* variants, suggesting that both diseases follow the same molecular pathogenesis. This observation was not surprising, considering that both *PSMD12* and *PSMC3* encode subunits that are constituents of the same protein complex, namely, the 19S RP within 26S proteasomes (Çetin et al., 2021). The notion of a causal link between variants of the 19S RP and NDDs with CHDs gained further support when one patient, afflicted with a neurological syndrome arising from mutations in the PSMC1 subunit of the 19S RP, was also found to have congenital heart malformations (Aharoni et al., 2022). As of today, all proteasome variants causing neurodevelopmental proteasomopathies are consistently found in genes encoding subunits of the 19S RP, specifically *PSMD12*, *PSMC1*, *PSMC3*, and *PSMC5* (Figure 1B) (Küry et al., 2017; Khalil et al., 2018; Aharoni et al., 2022; Ebstein et al., 2023; Küry et al., 2024).

The frequent association of CHDs with facial dysmorphism in neurodevelopmental proteasomopathies suggests a significant role of the 19S RP – and by extension, 26S proteasomes – in heart and craniofacial development. Several explanations might account for this connection, including the possibility that proteasome genes autonomously contribute to heart and brain development. Besides, given the expression of proteasomes in the placenta (Wang et al., 2013), it is also tempting to speculate that this dual phenotype may arise from defects in placental development. However, it is unlikely that impaired neurodevelopment is solely due to hypoxia caused by CHDs, as some patients with NDDs do not develop CHDs (Cuinat et al., 2023). One particularly compelling hypothesis suggests that the 26S proteasome dysfunction, which defines these disorders, may arise during the early stages of embryonic development, impacting specific cell populations that contribute to the formation of both organs.

## 3 Precursor cells in cardiac and neural development

**FIGURE 2:**
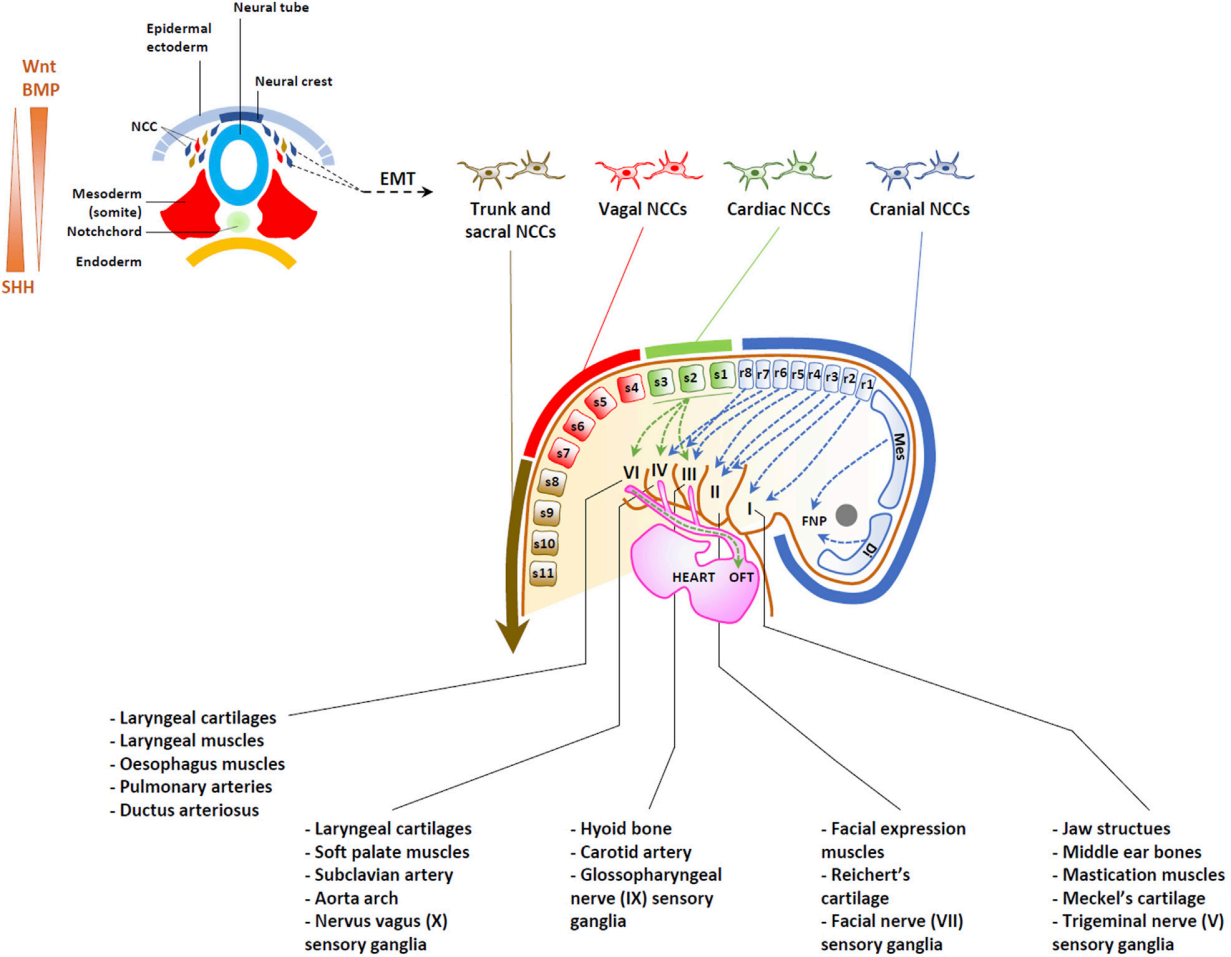
Origins of heart and head structures traced to neural crest cells (NCCs). Upper left: Dorsoventral representation of neurulation, depicting the generation of NCCs undergoing an epithelial-mesenchymal transition (EMT). BMP/Wnt and SHH act as morphogens, establishing opposing gradients within the neural tube, as indicated. Migrating NCCs can be categorized into cranial, cardiac, vagal, trunk and sacral NCC, depending on their position along the antero-posterior axis of the embryo, as indicated. While cranial NCCs from the diencephalon (Di) and mesencephalon (Mes) migrate into the developing frontonasal process (FNP), cranial NCC from the rhombomere levels (r) populate the pharyngeal arches I, II, III and IV to give rise to bones, cartilage, muscles, soft tissues and ganglia of most craniofacial structures. Cardiac NCCs arise from between the rhombencephalon and the third somite, migrating to populate pharyngeal arches III, IV, and VI, as well as the heart, contributing to the formation of the aortic arch and the septation of the future OFT.

Both the cardiac OFT and the craniofacial skeleton partially find their early origins in neural crest cells (NCCs), a multipotent migratory progenitor cell population derived from the ectodermal layer (McQuillen et al., 2010). The ectoderm is one of the three primary germ layers formed early in embryonic development and gives rise to various tissues and structures in the body, comprising the nervous system, the skin and the NCCs (Trounson, 2002). The process of embryonic development is highly orchestrated and involves a series of complex events, including migration, cell differentiation, tissue formation, and morphogenesis (Gerri et al., 2020). During early embryogenesis, the fertilized egg undergoes multiple cell divisions, leading to the formation of the blastocyst. The blastocyst subsequently differentiates into the three germ layers, one of which is the ectoderm. The dorsal ectoderm gives rise to the neural plate, which then folds and transforms into the neural tube. This process is known as neurulation (Copp et al., 2003). The neural tube will eventually differentiate into the brain and the spinal cord. As shown in Figure 2, as the neural tube forms, a group of multipotent cells referred to as NCCs emerge at its borders (Douarin et al., 2004).

Specifically, NCCs are assigned their identity through the fine-tune activity of several signaling pathways, among which bone morphogenetic protein (BMP), wingless-related (Wnt) factors, fibroblast growth factor (FGF), as well as neurogenic locus notch homolog protein (Notch)/Delta signaling interactions originating from neighboring regions including the neural plate, non-neural ectoderm, and mesoderm (Rogers et al., 2012; Alkobtawi et al., 2021). The early neural crest specifiers include the transcription factors FoxD3, Pax3/7, Ets1, Sox8/9/10, Twist1 and Snail1/2 (Schussler et al., 2021). The primary roles of the neural crest specifiers include three critical functions: 1) they establish NCC fate, 2) they initiate the epithelial-mesenchymal transition (EMT) process, characterized by the switch of cadherins, the secretion of extracellular matrix proteins such as laminin, fibronectin, collagen, vitronectin, thrombospondin, the activation of matrix metalloproteinases, the reorganization of the cytoskeleton, and the presence of distinct surface receptors including integrins and homing receptors, and 3) they play a role in preserving multipotency, by cooperation with pluripotency genes such as Nanog and Oct4 (Kirby and Hutson, 2010; Simões-Costa and Bronner, 2015; Scerbo and Monsoro-Burq, 2020; Zalc et al., 2021).

As the neural tube closes, NCCs begin migrating dorsolaterally under the surface ectoderm or ventrally along the neural tube (Theveneau and Mayor, 2012). As illustrated in Figure 2, NCCs can be categorized into four distinct subpopulations–cranial (sometimes referred to as cephalic), vagal, trunk and sacral–based on their axial origin along the anterior-posterior axis, spanning from head to tail (Milet and Monsoro-Burq, 2012; Simões-Costa and Bronner, 2015; Gandhi and Bronner, 2018). Cranial NCCs primarily reside anterior to the otic placode, extending across the forebrain, midbrain, and the anterior segments (or rhombomeres) of the hindbrain (Figure 2). The vagal NCCs, situated posterior to the cranial NCCs, are found at the levels of somites 1-7 and include cardiac NCCs (at the level of somites 1–3). Posteriorly, the vagal cell population is succeeded by trunk NCCs (somites 8–24) and sacral NCCs (somites 25–33).

More than thirty cell types are derived from NCCs in vertebrates. However, according to their axial level of origin, NCCs may display different potentials. From all levels, NCCs give rise to neurons and glia of the autonomic and sensory peripheral nervous system and to pigment cells progenitors. In addition, cranial NCCs provide mesenchymal precursors of most of head structures including bones, cartilage, connective tissue and tendons (Cordero et al., 2011). Moreover, crucial to our hypothesis, cranial NCCs also form the pericytes of forebrain blood vessels, which regulate the properties of the blood-brain-barrier (BBB) (Etchevers et al., 2001; Korn et al., 2002; Stebbins et al., 2019). Importantly, in addition to maintaining BBB integrity, capillary pericytes are believed to interact with various cell types besides endothelial cells, including astrocytes, microglia, and neurons, thereby participating in processes such as the regulation of neurogenesis (Zhou et al., 2022). In this regard, recent research has shown that pericyte dysfunction may impair neuronal differentiation by disrupting microglial function (Hattori et al., 2022). Furthermore, the notion that blood vessels play a role in brain development is supported by the observation that proper reelin signaling in endothelial cells is required for the development of cortical neurons (Segarra et al., 2018).

In contrast, cardiac NCCs actively contribute to the formation of the aorticopulmonary septum, heart valves and OFT (Figure 2) (Srinivasan and Toh, 2019). It has become clear in the last two decades, that a complex gene regulatory network controls the emergence of the neural crest fate, comprising both regulations common to all NCCs and transcriptional modules specific to NCCs subsets. This notion implies that during the early stages of development, NCCs destined to the heart or the head share signaling pathways, transcription factors, and other cell biology mechanisms that guide their specification, migration, and differentiation. This also suggests that any dysfunction affecting NCCs from all axial levels will have repercussions for both the heart and craniofacial development, as well as indirectly for brain formation via the activity of BBB. Understanding the shared developmental origins and pathways can provide valuable insights into the complex interactions between the heart and the head during embryogenesis and how disruptions in these processes can give rise to diverse clinical manifestations in affected individuals.

### 3.1 Cranial NCCs and their derivatives

The development of the head from cranial NCCs has been the subject of intense investigations over the past four decades in various animal models. It is understood that cranial NCC progenitors embark on their transcription program at the end of gastrulation, then during neurulation as well as several developmental stages prior to delamination and closure of the neural tube (Tan and Morriss-Kay, 1985). Controversy exists in the field regarding the mechanisms endowing these cells with such high levels of multipotency, with some studies suggesting a retention of pluripotency gene expression from blastula stages (Buitrago-Delgado et al., 2015; Pajanoja et al., 2023), while accumulating evidence proposes a reactivation of Oct4, Nanog, Klf4, and Sox2, during neurulation in NCC progenitors, sustaining the robust expression of the Twist1 and Prrx2 transcription factors (Scerbo and Monsoro-Burq, 2020; Zalc et al., 2021; Hovland et al., 2022).

In any case, cranial NCCs originating from the diencephalon and mesencephalon undergo dorsoventral migration, contributing to the formation of the frontonasal process (FNP) (Figure 2) as well as maxillary and mandibular processes (Kouskoura et al., 2011). By contrast, NCCs originating from rhombomeres 1 and 2 of the rhombencephalon migrate towards pharyngeal arch (PA) 1, giving rise to Meckel’s cartilage of the jaw, bones of the middle ear and the sensory ganglia of the trigeminal nerve (V). NCCs from rhombomere 4, and to a lesser extent rhombomeres 3 and 5 migrate towards PA2. Derivatives of PA2 include the hyoid and temporal bones, which arise from Reichert’s cartilage as well as muscles of facial expression and the sensory ganglia of the facial nerve (VII). Additionally, NCCs from rhombomeres 6-8 migrate primarily to PA3, while also making some contribution to PA4-6. These cells contribute to the development of laryngeal cartilage and muscles as well as the sensory glia of glossopharyngeal (IX) and vagus (X) nerves (Méndez-Maldonado et al., 2020) (Figure 2).

While the central nervous system (CNS) develops from the neural plate progenitors, emerging evidence indicates the involvement of NCC derivatives in CNS development. Early on, NCCs can act as sources for signaling molecules. Recent discussion by Bruet et al. (Bruet et al., 2023) has underscored abundant experimental data demonstrating that NCCs play a role in forebrain development by regulating crucial signaling pathways, notably by modulating FGF8 and Wnt activity along the dorsal midline. Moreover, as mentioned above, the function of the BBB, that regulates brain development, depends on the physiology of NCC-derived pericytes. It is also worth noting that, NCCs share common developmental events with the brain, including initial patterning of the anteroposterior axis, where any defects can impact both. Besides, the involvement of NCCs or NCC-derived structures in brain development is supported by the presence of intellectual disability traits observed in patients with established neurocristopathies, including chromosome 22q11 deletion syndrome (Cheung et al., 2014) and CHARGE syndrome (North et al., 1995). Indeed, these latter syndromes, along with several others, belong to a subset of CHDs that are associated with cognitive features and result from alterations in genes involved in NCC function (Vega-Lopez et al., 2018) (Table 1). Altogether, these observations suggest that defective NCC may disrupt brain networks and contribute to neurodevelopmental phenotypes.

### 3.2 Cardiac NCCs and their derived structures

As shown in Figure 2, cardiac NCCs populate PA 3-6, as well as the heart and great vessels, where they undergo differentiation into smooth muscle cells (SMCs). In these locations; they play a crucial role in the remodeling of the aortic arch, the septation of the OFT into the aorta and pulmonary arteries, and formation of the OFT valves (Ryckebüsch et al., 2010; Roux et al., 2017; Odelin et al., 2018; Stefanovic et al., 2021).

Migrating cardiac NCCs express high levels of MafB, Tbx2 and Tbx3 transcription factors (Tani-Matsuhana et al., 2018; De Bono et al., 2023), playing a pivotal role in heart development and contributing to the formation of the connective tissues of the thymus, thyroid and parathyroid glands (Monsoro-Burq, 2015). Recent work by De Bono et al. proposes that the specification of cardiac NCCs is governed by neighboring mesodermal cells expressing the transcription regulator Tbx1, which generates ligands, including Wnt and FGF, to drive the differentiation process (De Bono et al., 2023). Tbx1 also collaborates synergistically with SMAD7 in mesodermal cells to prevent premature activation of the TGFβ/BMP pathway, thereby preserving the expression of Tbx2 and Tbx3 in cardiac NCCs. Subsequently, progressive activation of the TGFβ/BMP pathway facilitates the acquisition of Gata3 and Isl1 markers, directing the differentiation of cardiac NCCs toward smooth muscle cells (SMCs) (Figure 2). In the context of heart development, cardiac NCCs facilitate the elongation OFT via incorporation of second heart field (SHF) mesoderm cells (Waldo et al., 2005) and participate in the septation of the aorta and pulmonary arteries (Waldo et al., 1998; Phillips et al., 2013).

## 4 Consequences of persistent proteasome loss of function

As discussed above, the co-occurrence of neurodevelopmental proteasomopathies with CHDs strongly suggests that the repercussions of proteasome dysfunction are particularly prominent within NCCs. A plausible rationale for the heightened susceptibility of NCCs to defective proteasomes might stem from the absence of fully adapted compensatory mechanisms. It is indeed plausible that the mechanisms could either be insufficient or overly robust, leading to diverse detrimental impacts on NCC physiology.

It is widely recognized that the physiology of NCCs depends on the proper functioning of the UPS. For instance, SoxE, an essential transcription factor guiding NCC differentiation into cartilage, glia, and melanocytes (Haldin and LaBonne, 2010), has been found to undergo modification by the ubiquitin-like protein SUMO (Taylor and Labonne, 2005; Lee et al., 2012). While the exact role of SUMO modification in this process remains incompletely understood, it is plausible that it could interfere with the ubiquitination process and thereby alter protein turnover, as previously described for the transcription factor MYC (Sun et al., 2021). Similarly, epithelial-to-mesenchymal transition (EMT) factors are recognized as proteasome substrates targeted for degradation by cullin-RING E3 ligases (Vernon and LaBonne, 2006; Lee et al., 2012).

Nonetheless, to the best of our knowledge, virtually nothing is known about the repercussions of proteasome dysfunction on the intricate series of events of NCC induction, specification, migration and/or cell fate determination. It can only be conjectured that the consequences of proteasome defects observed in other cellular or animal models might have implications for NCCs–and *a fortiori* in heart and head development–as well, as discussed below.

### 4.1 Disruption of protein homeostasis

Given the pivotal role of 26S proteasomes in the breakdown of intracellular proteins, their loss of function associated with neurodevelopmental proteasomopathies inevitably leads to increased accumulation of ubiquitin-modified proteins (Küry et al., 2017; Kröll-Hermi et al., 2020; Ebstein et al., 2023; Küry et al., 2024). This uncontrolled buildup of toxic proteins within the cytosol and nucleus has the potential to compromise cellular integrity and disrupt various vital biological processes, particularly intracellular transport (Wen et al., 2023). A large fraction of substrates continuously degraded by 26S proteasomes consists of defective ribosomal products (DRiPs), which are newly synthesized proteins that have failed to attain their final and native conformation following translation (Schubert et al., 2000). Notably, it is estimated that DRiPs constitute as much as 30% of all newly synthesized proteins (Yewdell and Nicchitta, 2006), suggesting that cells or tissues with elevated rates of protein synthesis face heightened degradation demands, rendering them more susceptible to the effects of 26S proteasome impairment. Interestingly, it seems that cranial NCCs proliferate at a slower rate than their cardiac counterparts (Ridenour et al., 2014), implying that precursor cells of the heart could accumulate a larger volume of protein aggregates than those of the brain under conditions of 26S proteasome impairment.

In addition to DRiPs, other substrates of the 26S proteasome include functional and long-lived proteins, that in response to specific signals, undergo ubiquitination for subsequent degradation. The removal of specific regulators by the UPS facilitates the modulation of diverse processes, enabling the cell to effectively adapt to its changing environment. In this context, the UPS regulates multiple signaling pathways including those involved in NCC induction, such as the BMP, Wnt, Notch, FGF, and Hippo transduction cascades (Zhu et al., 1999; Gupta-Rossi et al., 2001; Oberg et al., 2001; Wu et al., 2001; Wu et al., 2010; Zhao et al., 2003; Kowanetz et al., 2008; Voutsadakis, 2012; Li et al., 2014; Tu et al., 2014; Meng et al., 2016). Because 26S proteasomes may theoretically eliminate both positive and negative regulators of these pathways, the long-term effects exerted by defective 26S proteasomes on development may be difficult to predict. Nevertheless, it is widely acknowledged that inhibiting proteasome activity leads to diminished cell proliferation (Adams, 2004), thereby implying a potential decrease in the population of NCCs in embryos harboring proteasome loss-of-function variants.

In addition to their role in cell signaling, 26S proteasomes participate in the modulation of gene expression by degrading transcription factors and/or repressors. Consequently, sustained 26S proteasome dysfunction could lead to the stabilization of these factors. This aspect becomes particularly significant in cardiac development, given that certain DNA-binding proteins like ISL-1 need to be eliminated during the initial stages of heart development, especially within the OFT (Hatzistergos et al., 2020).

However, it is important to note that an analysis of the ubiquitination profile may not necessarily reflect the activity of the 20S proteasome, which can degrade disordered and/or oxidized proteins independent of ubiquitin (Abi Habib et al., 2020). It is indeed widely assumed that a non-negligible fraction of proteasomes within cells lack the 19S RP, allowing them to exist as free 20S proteasomes (Fabre et al., 2014). It is conceivable that alterations within the 19S RP could lead to disassembly of the 26S proteasome, thereby affecting the ratio of 26S to 20S proteasomes in patients with neurodevelopmental proteasomopathies. Future investigations will need to consider this and assess the quantity and activities of all proteasome complexes using a combination of native-PAGE and activity-based probes (ABPs) targeting the active sites of the threonine proteases, as previously described (Türker et al., 2023).

### 4.2 Increased autophagy

**FIGURE 3:**
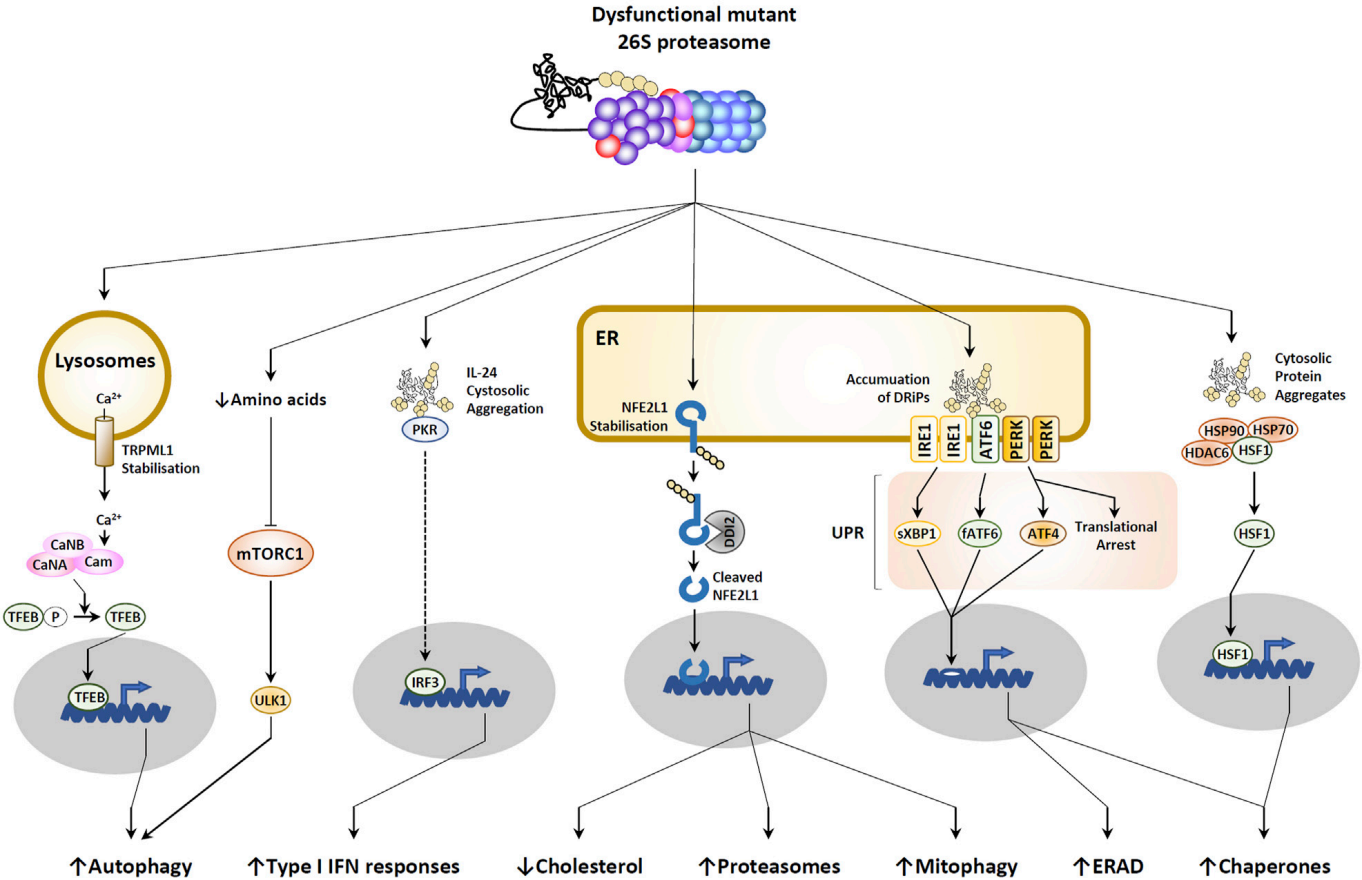
Consequences of proteasome dysfunction. Defective proteasomes lead to the accumulation of ubiquitin-modified proteins in the cytosol, resulting in the disassembly of the HSP90-HSP70-HDAC6-HSF1 complex and the subsequent initiation of a heat shock response, as indicated. Within these cytosolic aggregates, IL-24 is detected by PKR, prompting the induction of type I IFN responses. In lysosomes, increased calcium levels are linked to the stabilization of the calcium channel TRPML1. Autophagy activation is facilitated by decreased amino acid levels, leading to reduced mTORC1 signaling. In the endoplasmic reticulum (ER), the buildup of defective ribosomal products (DRiPs) triggers the unfolded protein response (UPR) through sensing by IRE1, ATF6, and PERK receptors. Stabilization of the ER-resident membrane protein NFE2L1 allows DDI2 protease to cleave it, releasing the C-terminal fragment that translocates into the nucleus to activate various programs (for detailed explanation, see text).

It is well established that proteasome dysfunction triggers a range of compensatory responses aimed at restoring protein homeostasis (Cuinat et al., 2023). As shown in Figure 3, not surprisingly, one of these programs is the autophagy-lysosomal degradation system, the second main degradation machinery in the cell (Kocaturk and Gozuacik, 2018). Indeed, by reducing protein breakdown and the subsequent availability of peptides and amino acids in the cell (Vabulas and Hartl, 2005; Suraweera et al., 2012), defective proteasomes activate autophagy by downregulating mTOR signaling (Feng et al., 2015). Additionally, there is an indication that the TRPML1 channel might also play a role in this phenomenon. Notably, as a substrate of the proteasome, TRPML1 becomes stabilized when proteasome function is compromised, subsequently promoting the release of calcium from lysosomes into the cytosol (Figure 3). The elevated calcium levels lead to the activation of a calmodulin-dependent phosphatase that dephosphorylates the transcription factor TFEB, enabling its nuclear translocation and the subsequent induction of autophagy-related genes such as SQSTM1, UVRAG, and VSP18 (Su and Wang, 2020) (Figure 3).

An ongoing debate revolves around the role of autophagy in NCC function. Initial studies suggested that excessive autophagy suppresses NCC survival (Wang et al., 2015); however, subsequent research has revealed that autophagy is indispensable for NCC induction (Wang et al., 2018). Moreover, autophagy could potentially influence cell fate determination, as the inhibition of autophagy may prompt cranial NCCs to adopt a chondrocyte fate (Yang et al., 2021). Interestingly, overactive chondrogenesis could result in delayed or impaired transition to bone, leading to underdeveloped or deformed facial bones. In any scenario, elevated autophagy should also lead to higher mitophagy rates, subsequently reducing the mitochondrial population within the cell. This aspect is particularly significant, as oxidative phosphorylation and its essential ATP production have been demonstrated to play a crucial role in the transcription of the NCC specifier FOXD3 (Costa et al., 2021).

### 4.3 Activation of the unfolded protein response (UPR)

Because the translocation of non-functional proteins from the ER lumen to the cytosol by the ER-associated degradation machinery (ERAD) is driven by proteasomes, any decline in proteasome activity results in protein burden within the ER compartment (Ebstein et al., 2019) (Figure 3). Consequently, this aggregation is sensed by the three ER membrane-resident IRE1, ATF6 and PERK receptors which in turn trigger the so-called unfolded protein response (UPR) by activating the downstream transcription factors sXBP1, fATF6 and ATF4 (Hetz, 2012) (Figure 3). The UPR is widely recognized as a stress response program designed to combat proteotoxic stress by increasing the expression of protein chaperones and transiently arresting translation (Ebstein et al., 2019). The stop in protein biosynthesis is mediated by phosphorylation of eiF2α by PERK, a PTM that blocks GDP/GTP exchange by eiF2B (Harding et al., 2000). Importantly, phosphorylation of eiF2α can be further supported by additional upstream kinases of the integrated stress response (ISR), including GCN2, PKR and HRI, which undergo activation following various cellular stresses such as amino acid depletion, viral infection or iron deficiency, respectively (Pakos-Zebrucka et al., 2016). Surprisingly, virtually nothing is known about the impact of sustained UPR in NCCs. However, the observation that GCN2 mutations are associated with pulmonary veno-occlusive disease (PVOD) (Eyries et al., 2014) suggests a critical role of this protein in cardiac development, a notion that remains, however, to be formally addressed. Besides, the impairment of stress response by mutations in *EIF2AK2* –which encodes a eiF2α kinase–and eiF2B genes–*EIF2B1* and *EIF2B2*– leads to leukoencephalopathy (Leegwater et al., 2001; Mao et al., 2020), hereby suggesting the importance of the UPR in CNS structure.

### 4.4 Activation of NFE2L1

Predictably, proteasome loss of function results in the stabilization of numerous proteins, including the ER membrane-resident protein NFE2L1 (also referred to as TCF11 or NRF1) (Radhakrishnan et al., 2010; Steffen et al., 2010). As shown in Figure 3, delayed degradation of this normally short-lived protein results in its cleavage by the protease DDI2 (Koizumi et al., 2016), whereby the C-terminal fragment enters into the nucleus to induce the transcription of genes coding for proteasome subunits and components of the mitophagy pathway (Radhakrishnan et al., 2010; Steffen et al., 2010; Yang et al., 2018) (Figure 3). Again, NFE2L1 processing upon proteasome impairment is regarded as a compensatory response destined to help the cell cope with proteotoxic stress by upregulating proteasomes and eliminating a potential source (i.e.,; mitochondria) of reactive oxygen species (ROS). Interestingly, once in the nucleus, NFE2L1 also inactivates LXR (Widenmaier et al., 2017), a transcription factor upregulating genes involved in cholesterol export such as ATP-binding cassette (ABC) transporters (Bilotta et al., 2020). This observation suggests that persistent proteasome dysfunction may lead to intracellular cholesterol deficiency (Figure 3). Considering the pivotal involvement of cholesterol in Sonic hedgehog (SHH) signaling (Xu and Tang, 2022), this line of reasoning could potentially signify a crucial function of NFE2L1 in the molecular underpinnings of NDDs associated with CHDs. Indeed, it has been demonstrated that SHH originating from the surrounding environment was a vital survival factor for both cranial and cardiac NCCs (Ahlgren and Bronner-Fraser, 1999; Arrigo and Lin, 2021).

### 4.5 Acquisition of type I IFN gene signatures

A few years ago, Goldbach-Mansky’s laboratory uncovered an unexpected association between proteasome pathogenic variants and the onset of sterile type I interferon (IFN) responses in patients afflicted with chronic atypical neutrophilic dermatosis with lipodystrophy and elevated temperature (CANDLE) (Brehm et al., 2015), a condition initially described by Torrelo and others (Torrelo et al., 2010). Since then, the causal relationship between proteasome dysfunction and the development of interferonopathies has been confirmed and validated by many other groups in various models (Poli et al., 2018; Zitvogel and Kroemer, 2021; Isidor et al., 2022; Yan et al., 2022; Ebstein et al., 2023; Waad Sadiq et al., 2023; Küry et al., 2024). The concept that cells carrying faulty proteasomes elicit a type I IFN response is intriguing, given that such responses typically arise during viral infections in response to pathogen-associated molecular patterns (PAMPs). A recent study conducted by Davidson et al. provided some clarity on this issue. Their work revealed that cytosolic aggregated IL-24 proteins function as a danger signal, activating protein kinase R (PKR) (Davidson et al., 2022) (Figure 3). This activation sets off a signaling cascade that ultimately leads to the production of type I IFN (Davidson et al., 2022). The precise rationale behind the production of type I IFN in response to disruptions in protein homeostasis remains elusive. However, its capacity to enhance the expression of immunoproteasomes and proteasome activators (Shin et al., 2007; Ebstein et al., 2009) suggests that it might serve the purpose of bolstering proteasome function to better manage proteotoxic stress. Currently, it remains unknown whether NCCs respond to proteasome dysfunction by generating IFNs, and even the capacity of NCCs to produce type I IFN is uncertain. This aspect warrants swift investigation, especially given that type I IFN has been demonstrated to adversely affect NCC migration (Pallocca et al., 2017), and more broadly, the maintenance of pluripotent stem cells (Eggenberger et al., 2019). The negative impact of type I IFN on development is further emphasized by the observation that prolonged IFN signaling has adverse consequences for cardiogenesis in individuals with Down syndrome (Chi et al., 2023) or neurogenesis in general (Zheng et al., 2014; Borsini et al., 2018; Kaneko et al., 2020). Collectively, these studies position type I IFN as a highly plausible disease driver candidate directly contributing to both heart and craniofacial malformations. However, this promising concept still needs to be substantiated from the perspective of NCCs.

### 4.6 Heat shock response (HSR)

It is also well established that malfunctioning proteasomes trigger a heat shock response (HSR) (Bush et al., 1997), albeit the underlying molecular mechanisms remain unresolved so far. As depicted in Figure 3, it appears that irrespective of temperature, proteotoxic stress prompts the disassembly of a high-molecular-weight cytosolic complex composed of HSP90, HDAC, and HSF1, thereby enabling the later to enter the nucleus and induce the transcription of specific target genes (Dai and Sampson, 2016). Not surprisingly, these genes encode protein chaperones including small heat shock proteins of the HSP family along with components of the UPS, collectively facilitating the alleviation of protein burden (Murray et al., 2004; Medicherla and Goldberg, 2008). Once more, the HSR in NCCs currently lacks documentation; nevertheless, the specific expression pattern of HSP during embryogenesis (Miller and Fort, 2018) as well as the upregulation of HSP27 and HSP47 in migrating NCCs (Rosenfeld et al., 2013; Liao et al., 2017) suggests that any disruption of this process could have significant implications for the development of the heart and head.

## 5 Prospective avenues for unraveling the molecular pathogenesis of neurodevelopmental proteasomopathies associated with CHDs

As highlighted repeatedly, our current understanding of proteasome biology and/or the regulatory mechanisms governing protein homeostasis in NCCs is ill-defined. Addressing this knowledge gap is, however, an essential prerequisite for unveiling the molecular pathogenesis underlying neurodevelopmental proteasomopathies associated with CHDs, as well as for contemplating therapeutic interventions. The reason for the lack of information on this subject is likely attributable to the constrained accessibility of NCCs from animal embryos resulting in a very limited amount of biologic material that is not suitable for conducting biochemical experiments.

However, this limitation can now be readily overcome by using human induced pluripotent stem cells (iPSCs). Indeed, in recent years, several studies have documented the successful generation of SOX10+ NCCs from iPSCs, often achieved through concurrent BMP inhibition and activation of Wnt signaling pathways (Menendez et al., 2011; 2013; Hackland et al., 2017). An ideal strategy to pinpoint the link between NDDs and CHDs would involve the further differentiation of iPSCs-derived NCCs into distinct cranial and cardiac NCC subtypes, each of which serving as precursor cells for craniofacial structures, pericytes and the OFT of the heart, (Stebbins et al., 2019). Of note, cranial identity can be established by adding BMP-4 during the process of NCC differentiation, which results in increased expression levels of cranial-specific DLX genes (Mimura et al., 2016). Unfortunately, as far as our current understanding goes, there is no existing protocol for generating cardiac NCCs from iPSCs. However, it has been demonstrated that supplementing iPSCs-derived NCCs with fetal bovine serum (FBS) and TGF-β results in the development of smooth muscle cells (SMCs) (Srinivasan and Toh, 2019) which participate in the heart OFT formation (Liu X. et al., 2019). In any case, deciphering the impact of proteasome loss-of-function variants on NCC biology would consist of 1) reprograming biological samples from patients with neurodevelopmental proteasomopathies into iPSCs before subsequent differentiation into various NCC subtypes or 2) introducing recurring loss-of-function proteasome mutations into control iPSCs by gene editing. The approach would then consist of determining the ability of these cells to differentiate into NCC and derivatives and uncover the impact of each of the compensatory pathways on this process.

## 6 Concluding remarks

The presented work highlights indeed a series of converging factors that point towards protein homeostasis disruptions within NCCs as a possible underlying cause for the concurrent emergence of NDDs and CHDs. Supporting this hypothesis, NCCs exhibit a particular vulnerability to pathogenic variants in ribosome genes commonly associated with ribosomopathies (Yelick and Trainor, 2015). This susceptibility suggests a high protein synthesis demand in NCCs, making them significant producers of potentially harmful DRiPs that require efficient clearance by the proteasome. Therefore, partial overlap in the clinical phenotype of neurodevelopmental proteasomopathies with certain ribosomopathies such as Treacher Collins syndrome (Vincent et al., 2016), Diamond-Blackfan anemia (Pallanti et al., 2008) or Roberts syndrome (Bermejo-Sánchez et al., 2011) is unsurprising. This overlap is particularly evident in craniofacial anomalies, cognitive impairment and, the frequency of cardiac malformations. While NCCs were discovered 150 years ago (Achilleos and Trainor, 2012), the acknowledgment of the vital significance of protein homeostasis regulation by the UPS is relatively recent, as underscored by the awarding of Nobel Prize in Chemistry to Aaron Ciechanover, Avram Hershko and Irwin Rose in 2004 (Behuliak et al., 2005). Although the exploration of this research field in NCCs is still in its early stages, its significance is expected to endure, given the pivotal role these cells play in the development of both the head and the heart. In this context, future research will have to assess NCCs for their equipment that preserve protein homeostasis, with the objective of understanding how disruptions of this equilibrium lead to the development of heart malformations and neurodevelopmental delay.

## Data Availability

The original contributions presented in the study are included in the article/supplementary material, further inquiries can be directed to the corresponding author.
